# Cell Interactions in Biliary Diseases: Clues from Pathophysiology and Repair Mechanisms to Foster Early Assessment

**DOI:** 10.3390/ijms20163961

**Published:** 2019-08-14

**Authors:** Luca Fabris, Carlo Spirli, Joachim Mertens

**Affiliations:** 1 Department of Molecular Medicine, University of Padua, 35131 Padua, Italy; 2 Liver Center and Section of Digestive Diseases, Department of Internal Medicine, Yale University, New Haven, CT 06519, USA; 3 Department of Gastroenterology and Hepatology, University Hospital Zürich, Rämistrasse 100, CH-8091 Zürich, Switzerland

## 1. Introduction

In modern hepatology, diseases of the biliary epithelium, currently termed cholangiopathies, represent one of the main gaps in knowledge, both on experimental and clinical grounds, though they started to draw attention since the late 80s [1]. Cholangiopathies comprise a large group of chronic liver disorders of many different etiologies, from genetic to immune-mediated (including the most common primary biliary cholangitis and primary sclerosing cholangitis), drug-induced, infectious, and ischemic, where the primary target of the damaging agent is the biliary epithelial cell, lining either the intra- or extrahepatic ducts [1,2]. Often thought as epidemiologically rare, cholangiopathies are instead relevant as a group, being the main indication of liver transplantation in the pediatric age (nearly 80%) and of a significant proportion of liver transplants in young adults (up to 20%) [1,2]. Clinically, cholangiopathies are characterized by a slowly evolving course, essentially marked by cholestasis; however, being largely asymptomatic, they remain difficult to detect for many years [1,2]. Notwithstanding, cholangiopathies may progress upon the effects of complex repair mechanisms relying on the cholangiocyte ability after injury to generate a multitude of signals gathering inflammatory, immune, and stromal cells into a highly reactive milieu [3]. Thus, in a translational perspective, cholangiopathies have become a model to understand liver repair and regeneration, and to delve into the pro-oncogenic potential of chronic inflammation. In this respect, the progression of primary sclerosing cholangitis (PSC) to cholangiocarcinoma (CCA) has been paradigmatic of this sequence. The aim of this book is to highlight some recent concepts on the variety of cell interactions, which, in cholangiopathies, are critical to sustain liver repair and regeneration, inflammation, and malignancy, and how they can provide diagnostic tools for the early assessment of cholestasis.

## 2. Cell Interactions in Liver Repair and Regeneration

Activation of the hepatic progenitor cell (HPC) compartment is a functional hallmark of ongoing liver injury, that, conversely from rodents, in humans, is triggered also by mild forms of damage. HPC are immature bipotent cells able to differentiate into both the biliary (through generation of ductular reactive cells) and hepatocyte (via intermediate hepatobiliary cells) lineages [3]. HPC are located in two specialized anatomical and functional niches—one is parenchymal, abutting the canals of Hering (HpSC), while the other is ductal, along the biliary tree (BTSC)—within the peribiliary glands (PBG) of the large intrahepatic and extrahepatic bile ducts. This topic is reviewed by Overi and colleagues [4], who describe the different engagement of the two compartments in human conditions, along with the niche cell composition and the specific signaling pathways underpinning their activation. Interestingly, HpSC activation and epithelial differentiation is finely regulated by the balance between Notch and Wnt ligands released by neighboring cells, such as portal myofibroblasts and macrophages. While HpSC activation is relevant in cholangiopathies associated with extensive fibrosis and disease progression, BTSCs are prominently involved in PSC upon the effect of Hedgehog ligands produced by the stromal cells localized nearby the PBGs and their degree of stimulation correlates with the progression of inflammation through dysplasia and cancer.

The role of PBGs as the cellular origin of CCA has been discussed by Nakagawa and colleagues [5], underlining their importance in inflammation-induced CCA (i.e., liver fluke infections) and in the intraductal papillary neoplasms of the bile duct. In this disease context, interleukin (IL)-33 (also known as “alarmin”) released by inflamed/damaged cholangiocytes behaves as a key mediator by inducing a potent type 2 innate lymphoid cell (ILC2) response, which in turn secretes IL-13 and amphiregulin, promoting cholangiocarcinogenesis from PBGs. IL-33 also stimulate a strong proliferative cascade mediated by IL-6/STAT3 signaling, a signature of the inflammation-associated cholangiocarcinogenesis. Interestingly, the IL-33-induced proliferative response was observed in cholangiocytes lining the extrahepatic but not the small intrahepatic bile ducts.

In addition to Notch, Wnt, and Hedgehog, yes-associated protein (YAP) is a morphogen instrumental for liver regeneration following cholestatic damage. In a mouse model of ductopenia induced by liver-specific deletion of the central downstream effector of Notch signaling, the recombination signal binding protein for immunoglobulin kappa j region (RBPJ), Tharehalli and coworkers found that YAP was activated in response to the increased expression of the scaffolding protein IQGAP1 induced by bile acid accumulation and stimulated a compensatory hepatocyte transdifferentiation towards the biliary lineage (intermediate hepatobiliary cells) with upregulation of the cholangiocyte marker SOX9 [6].

## 3. Cell Interactions in Inflammatory Cholangiopathies

Besides being the target of damaging insults of diverse etiologies, the cholangiocytes themselves may behave as initiators and propagators of the inflammatory and immune reactions [1,2,3]. This concept is well-exemplified in inflammatory cholangiopathies, where cholangiocytes undergo a range of phenotypic “neuroendocrine-like” changes enabling them to orchestrate the complex cellular and molecular interactions occurring in response to damage. Giordano and colleagues [7] addressed the pathophysiological role of gut-derived bacterial components or metabolites (including pathogen-associated molecular patterns, PAMPs) delivered to the hepatic parenchyma via the enterohepatic circulation in sustaining this “reactive” epithelial adaptation to injury of cholangiocytes. In this landscape, the authors emphasized the “leaky-gut” hypothesis, which has gained strong consideration in PSC based on the close association with inflammatory bowel diseases (IBD), mainly ulcerative colitis. However, the authors underlined how, in other conditions, as in the cystic fibrosis transmembrane conductance regulator (CFTR)-defective cholangiopathy, bacterial products (LPS) stimulate intense cholangiocyte secretion of pro-inflammatory cytokines via upregulation of TLR4 and the nuclear factor kappa-light-chain-enhancer of the *activated* B cells (NF-κB) pathway [8]. The intricate scenario of gut microbiota composition has been unraveled by several recent studies based on high-throughput technologies, in an attempt to define specific signatures in inflammatory cholangiopathies. Noteworthily, PSC patients seem to present a distinct microbiota profiling with enrichment in the *Enterococcus faecalis* species, regardless of its association with IBD [7].

The pro-oncogenic potential of a chronic fibroinflammatory microenvironment and the multiple molecular players regulating the reciprocal cell interactions have been reviewed by Cannito et al. [9]. The authors focused on two chronic cholangiopathies, namely PSC and congenital hepatic fibrosis/Caroli’s disease, heralding CCA. Experimental studies in both cholangiopathies have been favored by the availability of rodent models faithfully recapitulating the phenotype of biliary lesions. PSC progression to CCA has been linked to genetic mutations affecting p53 and KRAS signaling, as well as to nitric oxide production by the inflamed bile ducts. In a mouse model of congenital hepatic fibrosis, cholangiocytes harbored a perturbation of the β-catenin pathway regulating a low-grade inflammatory response mainly dominated by macrophages. However, further efforts will be needed to develop models reproducing the sequential steps underpinning cholangiocarcinogenesis in these cholangiopathies.

An important mediator of the abundant secretory functions enabling cholangiocytes to interact with other cell types is calcium (Ca^2+^) signaling. Rodrigues and colleagues [10] reviewed experimental models to study Ca^2+^ signaling in cholangiocytes and the Ca^2+^-dependent mechanisms regulating cholangiocyte secretory activity. The authors underline the role played by the inositol 1,4,5-triphosphate receptor (ITPR), the only intracellular Ca^2+^ release channel expressed by cholangiocytes, which is transcriptionally regulated by NF-κB and nuclear factor, erythroid 2-like 2 (NRF2), and activated by pro-inflammatory cytokines and oxidative stress. Importantly, impairment of Ca^2+^ signaling due to ITPR loss has been reported in a variety of cholangiopathies, including PSC, primary biliary cholangitis, and biliary atresia, and is responsible for ductular cholestasis. Thus, stimulation of Ca^2+^ may represent a useful strategy for the treatment of cholestatic conditions. 

## 4. Cell Interactions in Malignant Cholangiopathies

Cancer cells are embedded into a complex microenvironment, an “ecosystem” that constitutes an integral part of the “malignoma” and provides a multitude of factors to the growing tumor. The role of the stromal compartment and cell–cell interactions for the development of biliary malignancies has come into focus over the last years. In this highly desmoplastic type of cancer associated, activated stromal fibroblasts (CAF) often constitute the largest cell population, by far exceeding the number of actual cancer cells [11]. The clinical importance of CAF is supported by studies that show a negative correlation of CAF abundance and patient survival [12]. CAFs have been shown to alter and provide extracellular matrix components such as tenascin C and periostin, thereby facilitating cancer cell survival and migration. Moreover, they secrete growth factors such as platelet-derived growth factor (PDGF) that further support tumor growth and metastasis [13]. Therapeutically targeting this genetically homogeneous cell population is a novel concept [14], yet the cell interactions that contribute to biliary malignancy development and growth are far more complex as further cell types importantly contribute to the crosstalk. As Gentilini and colleagues describe comprehensively in their article [15], several subsets of immune cells are of importance for the process of malignant transformation. Probably in part attracted by activated CAF, an abundance of tumor-associated macrophages (TAMs) as well as reduced numbers of specific T-cell subpopulations have also been negatively correlated with patient survival. TAMs, a specific subset of alternatively activated so-called M2 macrophages, can promote cancer development by a number of mechanisms from growth factor secretion to matrix remodeling by metalloproteinases. Besides TAM, tumor infiltrating lymphocytes as well as NK cells may represent druggable targets in the microenvironment. While the stromal cells can be considered relatively genetically stable, the biliary cancer cells are characterized by an array of characteristic genetic aberrations. In their paper, Winkelmann et al. focus on the occurrence of microsatellite instability (MSI) in cholangiocarcinoma [16]. MSI has earlier been shown to be a favorable marker for therapy response, particularly to immune-modulatory treatments such as checkpoint inhibitors (CPI). As the authors demonstrate in a German cohort of CCA patients, MSI is a rare event in CCA, an important factor to consider when considering clinical trials of, e.g., CPI in CCA.

## 5. Cell Interactions Provide Tools for the Early Detection of Cholestasis in Cholangiopathies

Cholestasis is a fundamental feature of cholangiopathies, sustained by a complex interplay of several cell types contributing to biliary damage. Investigations to identify key biomarkers of the molecular mechanisms involved are sorely needed. As aforementioned, enterohepatic circulation, resulting in a bidirectional exchange of bioactive compounds, including endogenous (bile components) and exogenous metabolites (nutrients, xenobiotics, and gut bacteria) between the gut and the liver, is a central pathogenetic mechanism of the initiation and progression of cholestasis in several biliary disorders, particularly in PSC. By performing global metabolomic and lipidomic analysis in the portal serum and bile of patients with PSC, compared with noncholestatic chronic liver diseases, and of healthy controls, Tietz-Bogert and Kim et al. found that the bile in PSC had a unique and more homogeneous profile, enriched in dipeptides and polyamine metabolites, which can alter liver cell homeostasis as well as the intestinal microbiota [17]. Interestingly, polyamine catabolism has been associated with intense generation of reactive oxygen species, which represent an important determinant of disease progression in PSC.

Using real-time spectroscopic autofluorescence analysis of serum and liver tissue in experimental cholestasis (rats undergoing bile duct ligation), Croce and colleagues observed distinctive changes in some endogenous fluorophores. In serum, they found a significant increase in porphyrin derivatives, indicating perturbation of the heme metabolism, while in liver tissue, they described dramatic changes in the content of NAD(P)H, flavins, and lipofuscin-like lipopigments, reflecting mitochondrial dysfunction, oxidative stress, and accumulation of oxidized species [18]. Altogether, these molecular alterations may be regarded as chemical footprints of the initial events occurring in cholestasis, possibly to take advantage of for early diagnosis.

Oxidative stress is a main pathomechanism of reperfusion injury and biliary complications following liver transplantation. Cholangiocytes are indeed very sensitive to ischemic damage, leading to compensatory hyperplastic repair which mainly affects the large extrahepatic bile ducts, whereby it leads to biliary structuring. Schlegel and Dutkowski provided an overview on this topic, highlighting the protective effects exerted by machine liver perfusion [19]. In particular, the authors described how hypothermic oxygenated perfusion (HOPE) applied through the portal vein, eventually combines with the hepatic artery and protects cholangiocytes from ischemic injury, leading to a significant reduction in the posttransplant development of biliary strictures.

## 6. Conclusions

In conclusion, the papers compiled in this special issue underline the importance of different cell populations and their interaction in the development of biliary diseases. Much insight has been gained over the last few years in the different disease areas. Still, further in depth understanding and modelling of the cellular interactions is needed to move forward with the identification of druggable targets and for clinical trials that will hopefully soon benefit our patients suffering from hepatobiliary conditions.
